# Design of a Covert RFID Tag Network for Target Discovery and Target Information Routing

**DOI:** 10.3390/s111009242

**Published:** 2011-09-27

**Authors:** Qihe Pan, Ram M. Narayanan

**Affiliations:** Department of Electrical Engineering, The Pennsylvania State University, University Park, PA 16802, USA; E-Mail: qup100@psu.edu

**Keywords:** RFID tag network, pseudo-noise signal, cluster, routing

## Abstract

Radio frequency identification (RFID) tags are small electronic devices working in the radio frequency range. They use wireless radio communications to automatically identify objects or people without the need for line-of-sight or contact, and are widely used in inventory tracking, object location, environmental monitoring. This paper presents a design of a covert RFID tag network for target discovery and target information routing. In the design, a static or very slowly moving target in the field of RFID tags transmits a distinct pseudo-noise signal, and the RFID tags in the network collect the target information and route it to the command center. A map of each RFID tag’s location is saved at command center, which can determine where a RFID tag is located based on each RFID tag’s ID. We propose the target information collection method with target association and clustering, and we also propose the information routing algorithm within the RFID tag network. The design and operation of the proposed algorithms are illustrated through examples. Simulation results demonstrate the effectiveness of the design.

## 1. Introduction

RFID tags are small electronic devices working in the radio frequency (RF) range. They use wireless radio communications to automatically identify objects or people without the need for line-of-sight or contact, and have the advantage that they can be read through a variety of visually and environmentally challenging conditions. Their properties such as low cost, small size, and wireless functioning make them widely used in inventory tracking, object location, environmental monitoring, *etc*. Based on their energy source, RFID tags are categorized into three types: passive, semi-passive, and active. Passive RFID tags use energy from the incoming signal to power themselves, while semi-passive and active RFID tags use an internal power source, usually a small battery. Thus, active RFID tags can perform advanced functions and also work over longer ranges. RFID is an exciting area for research due to its relative novelty and exploding growth. Current research on RFID focuses on RF tags, readers, communication infrastructure, as well as some policy and security issues [1]. This paper explores the applications of active RFID tags in target identification in the RFID tag network using a pseudo-noise signal, where a static or slowly moving target out of the range of the command center transmits a distinct pseudo-noise signal within the field of the spatially distributed RFID tags. These RFID tags in the network collect the target’s information and route it to the command center. The noise signal from the target is known only to the RFID tags in the network so they can easily detect it. However, this signal is not detected by undesired parties since the transmitted signal has unpredictable random-like behavior and does not possess repeatable features for signal identification purposes [2]. Noisy tags, which are regular RFID tags that generate noise, can be used to help establish a secure channel between the reader and the queried tag. A noisy tag protocol is proposed in [3], wherein a noisy tag in the reader’s field sends out a noise signal generated from a pseudo-random function, the secret shared with the reader. The reader can reconstruct and subtract the noise signals from the noisy tag and recover the message from the queried tag, while an eavesdropper is unable recover the queried tag’s message. An eavesdropping-resistant and privacy-friendly RFID system is developed in [4], in which the chip modulates its reply onto a noisy carrier provided by the reader to protect the back-channel against eavesdropping. This method does not require additional protective devices.

Cluster approaches have been used a lot in parametric frameworks for detection and estimation. Sensors are partitioned into subgroups for distributed learning in the wireless networks [5]. In addition, the cluster approach is also used for topology control [6]. In this paper, we employ RFID tag clusters within the RFID tag network to collect the target’s information. There are two steps in this process: (1) target association; and (2) cluster formation and cluster head selection. If an RFID tag detects the target, then it stores the target’s ID and gets associated with the target. Clusters are formed by RFID tags associated with the same target. One of these RFID tags, selected as the head of the RFID tag cluster, routes the target’s information out to the command center. In our design, the RFID tag with the maximum number of links to the outside of the cluster is selected as cluster head, which is robust to channel failures, considering that the RFID tags in the network are battery driven and may run out of life. When some of the communication links between the cluster head and those RFID tags out of the cluster are broken, the cluster head RFID tag still can use alternate communication links between it and RFID tags outside of the cluster to route the target’s information out.

There are many approaches for information routing in the wireless sensor networks from different aspects of view. In [7], an information-directed routing method is proposed for localization and tracking problems, in which routing is formulated as a joint optimization of data transport and information aggregation, and information accumulated is maximized along the routing path. In [8], selection of the set of cluster heads is defined as the weighted connected dominating set problem, and centralized approximation algorithms are developed to select them. A maximum energy welfare algorithm is designed in [9] by applying the social welfare functions to the routing in wireless sensor networks. Each sensor makes routing decisions to maximize the energy welfare of its local society, which leads to globally efficient energy-balancing due to overlapping of the local societies. RFID tags can also be used to route information in the networks. In [10], the active relay tags retransmit their received signals during the communication between the interrogator and active tags, and the proposed RFID multi-hop relay system can achieve larger coverage. In our approach, the routing path in the RFID tag network from cluster head RFID tag to the command center is selected according to the channel condition, which is a joint optimization of favorable channel conditions and short path length. Each RFID tag intelligently selects its successor and routes the target’s information to it. There are two stages when each node selects its successor on the routing path based on two criteria: (1) channel quality sensing; and (2) target’s information routing. During channel quality sensing, the channel condition is estimated and quantized to form the link weight, while in the information routing stage, the RFID tag determines its successor based on the channel information obtained and sends the target’s information to it.

In this paper, we present an algorithm design in the physical layer on target information collection and routing within the RFID tag network in outdoor scenarios, specify the signal format, signal modulation, and signal detection method. Using a noise signal as the information carrier and a noisy key at the front of the RFID tag’s signal indicating the purpose of the message, guarantees that the communication within the RFID tag network is covert, owing to the low probability of interception and low probability of detection of the noise waveform. During the RFID tag cluster head selection process, the RFID tag with the maximum number of links to the outside of the cluster is selected as the tag cluster head, and it routes the target information out to the command center. The RFID tag cluster head selected in this manner is robust to channel failures. When some of the communication links between it and the RFID tags out of the cluster turn down, which may occur due to the battery failure in those RFID tags, it still can use the other communication links between it and RFID tags outside of the cluster to route the target’s information out. The routing path from RFID tag cluster head to the command center in the RFID tag network we propose is based on the joint optimization of channel quality and path length.

The rest of the paper is organized as follows. Section 2 gives the design of the RFID tag network and procedures for target’s information collection in the RFID tag network. Section 3 presents the algorithm for target’s information routing within the RFID tag network, and it is illustrated through examples. In Section 4, we discuss implementation issues for hardware realization. Section 5 draws the conclusions of this article and presents possible future extensions.

## 2. Target Information Collection

**Figure 1.**
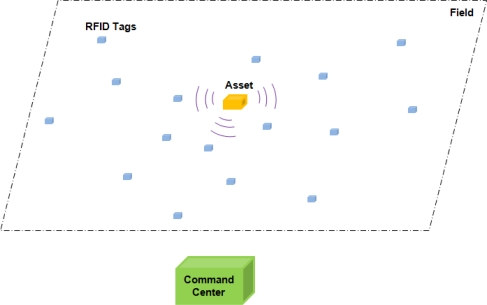
RFID tag application scenario.

The application scenario is depicted in Figure 1. A static or slowly moving target is in the field of RFID tags, out of the range of the command center. The cooperative target transmits a distinct RF pseudo-noise signal. The goal of the RFID tag network design is to collect the target’s information and route it to the command center with assistance of the deployed RFID tags. The RFID tags here do not know their own locations. A map of their locations is saved at the command center, so the command center can determine where a RFID tag is located based on the RFID tag’s ID.

In this stage, there are two steps for collecting the target’s information: (1) target association; and (2) cluster formation and cluster head selection. In target association, some of the RFID tags detect the target by sensing the environment and record the target’s information, and thus these RFID tags are associated with that target. RFID tags associated with the same target form tag clusters. Within a cluster, RFID tags share the same information associated with the target, so when some of the RFID tags turn down due to battery failure, *etc*., other RFID tags still have the target’s information. One RFID tag, namely the head of the RFID tag cluster needs to route the target’s information out. The cluster head RFID tag is chosen during cluster head selection.

### 2.1. Target Association

The target under monitoring transmits its distinct signal in noise form in the RFID tag field. The RFID tags designed here have templates of the signals from possible targets of interest. They listen to the environment and detect whether there is any target that is on the monitor list of potential targets. Each RFID tag recognizes the target by comparing its received signal with its template signals in its memory. Once an RFID tag detects a target, it records the target’s information, such as the target’s ID. Since signal transmitted by the target is of random noise, RFID tags use the cross-correlation process to determine whether the target exists in the field or not. In the real world, the environment is more complex with various interferences such as clutter, Doppler shifts, *etc*., which are not fully discussed here since they are not the main focus of the paper.

Suppose the target transmits pseudo-random noise signal burst *s*(*t*) over time *T*_0_, and RFID tag has the library of signals from possible targets on the list {*s_i_* (*t*)},*i*= 1, 2,...,*M*, where *M* is the number of targets in the monitoring list. That is, in the RFID tag’s library, signal *s_i_*(*t*) is a template of the signal transmitted by the *i*^th^ target. The detection output at the RFID tag is:

(1) $$\mathrm{corr}\left(\tau \right)=\int_{0}^{T}s\left(t\right)s_{i}\;\left(t-\tau \right)dt,\;\;\;\mathrm{for}\;j=1,2,\ldots ,M$$

If there is a peak at some time index of the correlation output, it means that the target’s signal does exist, and therefore the target’s ID is determined.

**Figure 2.**
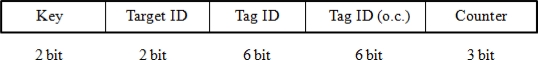
RFID tag’s signal general format.

The RFID tag records the ID of the target that it is associated with to its memory variable *flag_tag_id_*, and modulates it to the tag’s signal. By default, if an RFID tag is not associated with any target, its *flag_tag_id_* is 0. The RFID tag’s signal has the general format shown below (Figure 2), where each section is denoted by the bits under it. For each section, the all-0 bit message means that the RFID tag’s signal contains no specific information of that section.

Each RFID tag’s base signal comes from filtering a band-limited pseudo-noise signal *s_tag_*(*t*) to a specified and unique frequency sub-band. Different RFID tags have non-overlapping frequency bands, and they all have knowledge of *s_tag_*(*t*) in advance. For example, RFID tag *k*’s base signal *s_tag_kb_*(*t*) is *s_tag_*(*t*) filtered to its *k*^th^ sub-band, and RFID tag *k*’s signal *s_tag_k_* (*t*) modulated with message is described as:

(2) $$s_{\mathrm{tag\_k}}\;\left(t\right)=\sum_{n=0}^{L-1}a_{n}s_{\mathrm{tag\_kb}}\;\left(t-\mathrm{nT}\right)$$

where *T* is the time duration of *s_tag_kb_*(*t*), *n* is the index of the bit, *a_n_* is the valued of the *n*^th^ bit, and *L* is the number of total bits of RFID tag’s signal.

**Figure 3.**
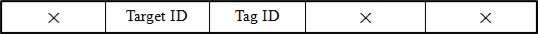
RFID tag’s signal format after association with a target.

After association with a target, the RFID tag’s signal has the format shown in Figure 3, where Target ID denotes the target’s ID that the RFID tag is associated with.

If a target is in the field, only a subset of the RFID tags can collect its information. This is due to the fact that the distance between the target and the RFID tags may be larger than the detection range of some of the tags, or that the channel condition is very bad due to excessive noise making the error probability from that link above the tolerance level.

**Figure 4.**
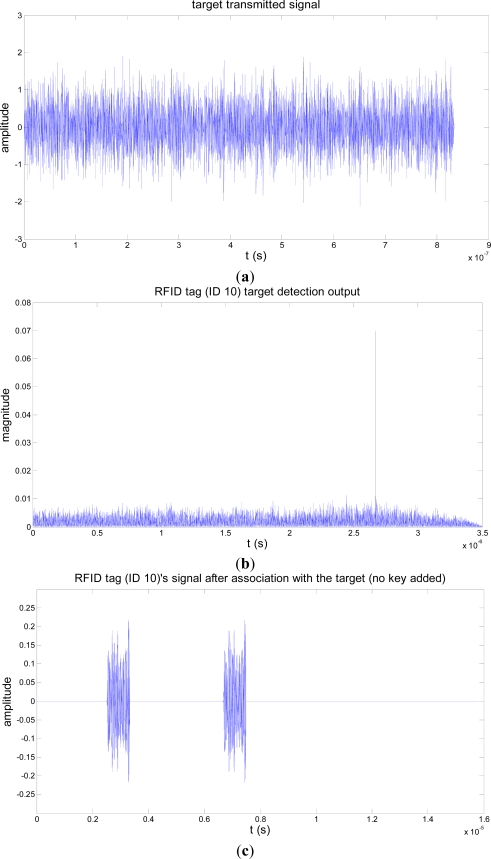
Target association simulation illustration. (**a**) noise signal transmitted by the target; (**b**) correlation output of RFID tag (ID 10) indicating target detection; (**c**) RFID tag (ID 10)’s signal after association with a target with no key.

The target association process of an RFID tag is illustrated with simulations in Figure 4. In the simulation, the target’s signal is assumed to be over the 1–2 GHz frequency band. We assume also that there are 50 RFID tags in the field, the pseudo-noise signal *s_tag_*(*t*) is over 1–2 GHz, and the RFID tag (ID 10)’s signal is over the 1–1.0187 GHz sub-band. RFID tag (ID 10) detects and gets associated with the target (ID 01) in a channel with a signal-to-noise ratio (SNR) of −3 dB. The negative SNR shows that the target association process is performed covertly since the signal power is less than that of the channel noise.

### 2.2. Cluster Formation and Cluster Head Selection

RFID tags associated with the same target are formed as a cluster. In each cluster, cluster head RFID tag is selected through inter-communication among the cluster member RFID tags, and is responsible for routing the target’s information the cluster’s associated with to the outside of the cluster. Using cluster head RFID tag to route the target’s information out is to reduce the information redundancy and signal interferences from multiple RFID tags.

RFID tags here are power driven devices, so the links between them may fail occasionally. To ensure connectivity, the RFID tag with the maximum number of links to the outside of the cluster is selected as cluster head, which is responsible for routing the target’s information it carries to the outside of the cluster. The cluster head RFID tag selected accordingly is robust to channel failures. When some of the communication links between it and those RFID tags out of the cluster turn down, the cluster head RFID tag still can use the other communication links between it and RFID tags outside of the cluster to route the target’s information out. When a tie occurs, *i.e.*, when two or more concurrent RFID tags have the same number of links to the outside of the cluster, the one with the highest energy level is selected as the cluster head.

We model the RFID tag network as a graph *G*(*V*,*E*), where *V* is the set of nodes in the graph *G*, and *E* is the set of edges. The cluster of RFID tags is modeled as a subgraph *C* of *G*. Each RFID tag is represented by a node in the graph and the communication channel between RFID tags is represented by an edge. Then, the cluster head RFID tag is the start node on the routing path of the target’s information with which all RFID tags in the cluster are associated.

In our system, the RFID tag is designed to operate in two modes. In Mode I, the default mode, the RFID tag works at normal energy level. In Mode II, the RFID tag works at higher energy and has longer communication distance. Most of the time, the RFID tags operate in Mode I. In the case an RFID tag needs a larger range, for example, when it tries to find its neighbors but cannot find any in the default mode, the RFID tag will go to Mode II. When the task is finished, the RFID tag will return to the low-energy Mode I.

For the design of the RFID tag’s operating Mode II, we assume that each cluster of RFID tags is a connected component in the RFID tag network. That is, for each pair of nodes *u*, *v* ∈ *V*(*C*), there is a *u*,*v* -path in *C*. Thus, RFID tags within the same cluster are able to get messages of the rest in the cluster tags. From these messages, the RFID tags recognize other member RFID tags in their cluster and the cluster head RFID tag is determined.

**Figure 5.**
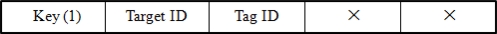
RFID tag’s inquiry signal format for counting outside links.

After sensing and association with the target, the RFID tag starts to discover and count its links with RFID tags not associated with the target. The RFID tag associated with the target sends out its outside-link sensing signal of the format shown in Figure 5.

The Key at the front of the RFID tag’s message is globally defined, known by all the RFID tags in the field, to indicate the purpose of the RFID tag’s message. Here, Key (1) indicates that the RFID tag which sends out the message is sensing and counting its links with RFID tags outside the cluster. For signal covertness, the Key is designed to be a noise waveform. RFID tags in the network have templates of the Keys, and they can recognize the corresponding Keys by cross-correlating the incoming signal with their stored templates of Keys.

**Figure 6.**
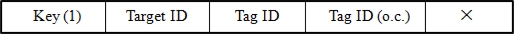
RFID tag’s response signal format for counting outside links.

If an RFID tag hears the link inquiry from one RFID tag within the cluster, it obtains the Key in the message and determines the type of the Key. If the Key is Type 1, it decodes the message to get the target’s ID. In addition, it checks whether its own signal has that particular target’s ID stored. If it is not associated with the target, it sends back the signal modulated with its ID; else, it does not respond. This guarantees that the RFID tag in the cluster only counts its links with RFID tags outside the cluster. The RFID tag outside the cluster responds to the cluster member RFID tag with the signal format shown in Figure 6 upon the link counting inquiry, where Tag ID (o.c.) denotes the RFID tag’s ID outside the cluster.

**Figure 7.**
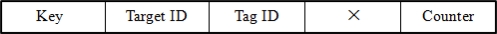
RFID tag’s signal format after searching for links to the outside of the cluster.

The RFID tag stores the number of its links with RFID tags outside the cluster in a counter, which is set to zero (0) by default. RFID tag sensing links with those outside the cluster obtains and determines whether the Key in the message is Type 1 upon its received signal, if so, it decodes the message. It checks whether the first Tag ID in the message is the same as its own to determine whether the message is a response to its link counting inquiry. Then it increases the number of links in its counter by 1 if there is a new RFID tag ID in the message. After searching for links to the outside of the cluster, the RFID tag updates its signal following the format in Figure 7, where Counter saves the number of links the RFID tag has to the outside of the cluster, and Key is set to initial value which is blank and has no meaning about the function of the message.

After time Δ*t*_1_ from the time it sends out the link counting inquiry signal, the RFID tag stops receiving the responses to its link counting inquiry, and finishes counting the number of links it has with RFID tags outside the cluster. An approximate Δ*t*_1_ is given as:

(3) $${\Delta t}_{1}\approx \frac{2R}{c}$$

where *R* is the range of RFID tag, and *c* is the speed of light in the air.

If the RFID tag cannot find any neighbor outside the cluster at this time, it changes to operation Mode II, and starts searching the links again. In Mode II, the RFID tag functions with more energy than in the default mode. With the design of Mode II, we assume that at least one RFID tag in the cluster has positive Counter. The RFID tag returns to the default operation mode after completing searching its links to the outside of the cluster.

**Figure 8.**
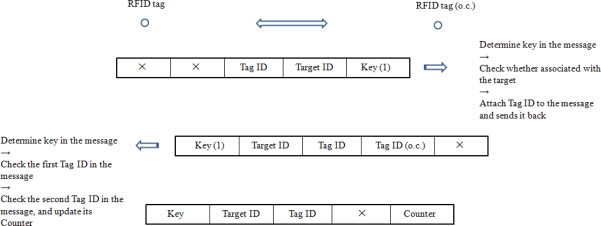
Link counting process of RFID tag in the cluster.

The RFID tags in the cluster that complete the whole searching for links in Mode I wait for time Δ*t*_1_ from the time they finish searching for links. Thus, all the RFID tags in the cluster spend the same time 2Δ*t*_1_ on the process to search for links to the outside of the cluster. The link counting process for RFID tag associated with a target is depicted in Figure 8.

Some of the RFID tags in the cluster may be closer to the target than others, so they send out the link count inquiry signals earlier, and complete the link searching and counting process described earlier. We set a time variable Δ*t_w_*[*tag_id*] for each RFID tag to wait after it completes the link searching process, before starting inter-cluster communication. Thus, when the RFID tags in the cluster start inter-cluster communication, they have all finished counting the links and each of their Counters stores the final values.

An approximate value for Δ*t_w_*[*tag_id*] is as follows:

(4) $${\Delta t}_{w}\left[\mathrm{tag\_id}\right]\approx \frac{K}{t_{T}\left[\mathrm{tag\_id}\right]}$$

where *K* is a constant, and *t_T_*[*tag_id*] is the target discover time at that RFID tag. Thus, the RFID tag closer to the target wait for longer time after complete searching and counting its links to the outside of the cluster.

**Figure 9.**
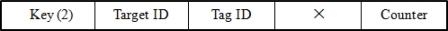
RFID tag’s format for inter-cluster communication.

Then the RFID tags in the cluster starts inter-cluster communication to recognize the members in the cluster and select the cluster head RFID tag. Each RFID tag broadcasts its signal in the format shown in Figure 9, where Key 2 indicates that the message is communicated among RFID tags in the cluster to select the cluster head.

Upon receiving the signal, the RFID tag in the cluster obtains the Key in the message and determines whether it is Key 2. If it is Key 2, the RFID tags with their *flag_tag_id_* registered will involve in the inter-cluster communication, and those with *flag_tag_id_* of 0 will not. In the case there is only one target in the field, this also indicates that the RFID tag is within the same cluster. In the complex case of multiple targets in the field, the RFID tag needs to further check the Target ID in the message to determine if it is the same as its own or not. If so, the message is from an RFID tag in the same cluster. After determining that the message is from an RFID tag in the same cluster, the RFID tag continues to decode the message and checks whether the Tag ID in the message is the same as its own. If the message is from another RFID tag for the purpose of cluster head RFID tag selection, it forwards the message and compares the Counter in the message with its own. If the Counter in the message is larger than its own, the RFID tag sets its own Counter to −1, which indicates that its number of links to the outside of the cluster has been compared and not the largest.

After sufficient time Δ*t*_2_, each RFID tag in the cluster completes deciding whether it has the most number of links to the outside of the cluster. The RFID tag whose Counter is positive will become the cluster head, and it then starts to route the target’s information out. Then all the RFID tags’ Counters will be initialized to zero.

**Figure 10.**
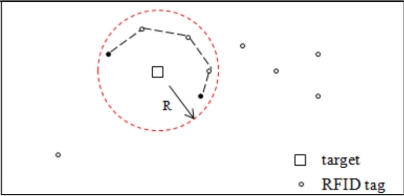
Case for maximum Δ*t*_2_.

An approximation for Δ*t*_2_ is given as follows:

(5) $$\Delta t_{2}=\frac{2\pi R-R}{c}$$

which is a little larger than the worst time of cluster head selection. This approximation for Δ*t*_2_ in Equation (5) is based on the case shown in Figure 10.

As stated before, the RFID tag network is modeled as a graph, where each node represents an RFID tag and each link represents the communication link between RFID tags. The cluster of RFID tags is then a subgraph, and it is a connected component in our assumption with the design of RFID tag’s operation Mode II. In the case shown in Figure 10, the two black nodes are in the same cluster, but the distance between them exceeds their range, and they cannot communicate with each other directly. Since the cluster is a connected component, there exists a path in the cluster connecting the two nodes. Through message forwarding by other nodes in the cluster, the two black nodes can communicate indirectly, for example, following the route in dashed line in Figure 10. The route length is on the order of 2*πR*–*R*, and this costs time on the order of $\frac{2\pi R-R}{c}$. Thus, the cluster head is the RFID tag with the maximum number of links to the outside of the cluster.

In a complex case, several RFID tags in the cluster have the same number of links to the outside of the cluster. Since each RFID tag is ignorant of its location, it does not know whether it is nearest to the command center or not. Thus, the RFID tags in the cluster are unable to select the one nearest to the command center among them as the cluster head. Instead, they may further communicate to select the one with most energy as the cluster head. In this paper, we restrict the situation to the simple case that there is no tie.

After a short time, when the target’s information is routed to RFID tags that have no links with the RFID tags in the cluster, RFID tags in the cluster set their *flag_tag_id_* to 0, return to the beginning state and starts a new cycle. They perform target association, cluster formation, and cluster head selection again.

**Figure 11.**
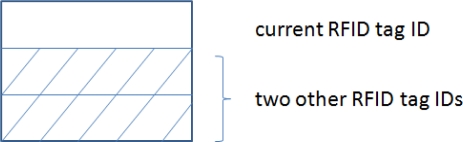
Signal format modification for target location determination.

Additionally, if we upgrade the RFID tag design, as shown in Figure 11, such that the cluster head RFID tag is capable of saving the IDs of other RFID tags in the cluster during the inter-cluster communication, and it incorporates that information to the message to be routed outside the cluster, the target’s location can also be determined at the command center. As stated before, the command center has a map of all the RFID tags. Thus, if the IDs of at least three RFID tags associated with the target are known, the locations of these three RFID tags are known at the command center, and thereby the location of the target can be determined.

## 3. Information Routing

The signal sent by an RFID tag is not of very high power. With the assumption that the communication cost is proportional to the communication distance, the goal of information routing in the RFID tag network is to select a channel which is robust and of short path length to route the target’s information gathered by the RFID tags to the command center. When each node selects its successor on the routing path, there are two stages during the process: channel quality sensing and target’s information routing. In channel quality sensing, the link weight is estimated based on the corresponding channel condition. In the information routing stage, the node determines its successor and sends the target’s information to it.

**Figure 12.**
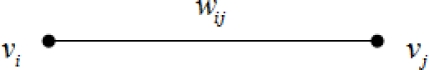
Model of a link.

The RFID tag network is modeled as a two dimensional graph *G* = (*V*,*E*), where *V* = {*v*_1_,*v*_2_,. . ., *v_n_*} is set of the nodes, representing the RFID tags, and *E* is the set of bidirectional links, representing the communication links between RFID tags. Each link, shown in Figure 12, is assigned a positive weight which indicates the robustness of its corresponding communication channel. If the quality of the communication channel of is good, its weight is small; if the channel is bad, for example, very low SNR, excessive fading, object blocked channel, *etc*., its weight is very large; if there is no link between the two nodes, the weight is ∞. The weight for link (*v_i_*, *v_j_*) is expressed as *w_ij_*.

### 3.1. Channel Quality Sensing

**Figure 13.**
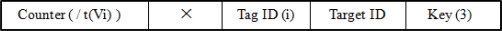
RFID tag’s signal format for channel sensing.

In this step, the RFID tag senses the channels. All its neighbor RFID tags calculate the weights of links connected to them based on their received signals, and respond to the RFID tag with updated messages. If a tag does not find any neighbor, it transmits in power Mode II. The RFID tag sends out the signal format shown in Figure 13 for channel sensing, where Key (3) indicates that the message is for channel sensing.

**Figure 14.**
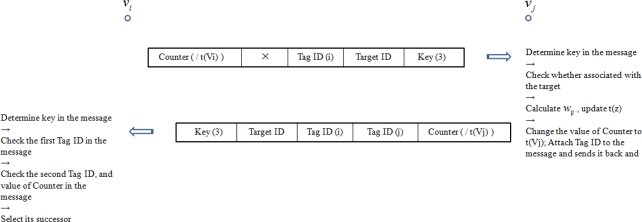
Channel quality sensing process between two RFID tag nodes.

The channel quality sensing process between two RFID tag nodes is depicted in Figure 14. Since RFID tags in the cluster associated with the target have the target ID stored in their memory variables *flag_tag_id_*, when they receive the channel quality sensing message indicated by Key (3), they will not respond and thus will not be involved in the target’s information routing. As for RFID tags outside the cluster, their memory variables *flag_tag_id_* do not have the target’s ID, and they will participate in routing the target’s information.

In Figure 14, node *v_j_* decodes the signal from node *v_i_*, estimates the channel condition for link (*v_i_*,*v_j_*), quantizes it, and grades it to the link weight *w_ij_*, generated by a channel quality quantization function. The value of Counter in the message is *t*(*v_x_*), where *x*=*i, j*. Also, *t*(*v*), *v* ∈ *V*(*G*) is defined in the information routing section below. The estimation of channel information from the received signal is outside the scope of this paper. Several papers have discussed this issue, e.g., [11].

The channel condition is quantized and graded to several statuses at the RFID tag, denoted by the weight of the link. The channel quality quantization function may be based on the SNR of the channel, for example, as an inverse function of it. The channel quantization is also not a main concern for discussion in this paper, and the details are not presented here. Good channel quality is quantized to small link weight, while bad channel quality is quantized to large link weight. An upper limit value *w*_max_ for the link weight is set. If the link weight is larger than *w*_max_, there is no link between the two nodes or the link between the two nodes is not usable.

### 3.2. Information Routing

The channel aware information routing in the RFID tag network is to find a shortest path with good quality channels from the cluster head RFID tag to the command center. Since the range of RFID tags is not very large, the length of each hop does not vary much. We model the length of the path as the number of hops from a node to the command center. Then routing problem then turns into an optimization problem as follows:

(6) $$\text{min}\;\text{cost}=\left(\sum_{i,j\in \left\{1,2,\ldots ,N\right\}}w_{\mathrm{ij}}x_{\mathrm{ij}}+\sum_{i,j\in \left\{1,2,\ldots ,N\right\}}x_{\mathrm{ij}}\right),\;\text{where }\;x_{\mathrm{ij}}=\{\begin{array}{c} 0\\ 1 \end{array}$$

The first term of the equation denotes the channel condition of each hop. If the channel of the hop is good, then the weight *w_i_* assigned to that link is small. The second term of the equation denotes the length of the path. Thus, Equation (6) is used to find a routing path with both good link quality and short length.

Equation (6) is equivalent to:

(7) $$\text{min}\;\text{cost}=\sum_{i,j\in \left\{1,2,\ldots ,N\right\}}w_{\mathrm{ij}}x_{\mathrm{ij}}$$

since the two terms are independent of each other.

Equation (7) can be solved using Dijkstra’s Algorithm [12]. Given a graph with nonnegative weights and a starting node, Dijkstra’s algorithm finds the shortest path from the starting node to other nodes in the graph. Its basic procedure is:

**Table:** 

Starting node: *u*, weights of edges *w_ij_*, *i*, *j* ∈ {1,2,...,*N*}
Initialization: *S* = {*u*}, *t*(*u*) = 0, *t*(*z*) = *w_uz_* for *z* ≠ *u*
Iteration: Step 1: select a node *v* ∉ *S* such that *t*(*v*)=min_*z*∉*S*_*t*(*z*); *S* = *S* ∪{*v*}
Step 2: for each edge *vz* with *z* ∉ *S*, *t*(*z*) =min{*t*(*z*),*t*(*v*)+*w_vz_*}
Iteration continues until *S* = *V*(*G*) or *t*(*z*) = ∞ for each *z*∉*S*
Length of shortest path between nodes *u*, *v*, is *d*(*u*,*v*) = *t*(*v*) for all *v*.

The stopping rule of the iteration in our algorithm is modified to *v* = *destination* or *t*(*z*) = ∞ for each *z* ∉ *S*.

As for routing through holes, many papers have discussed this issue, such as [13,14]. Some complete void handling techniques include Greedy Perimeter Stateless Routing (GPSR) [15], Distance Upgrading Algorithm (DUA) [16], *etc*. We did not expand it here.

We illustrate the operation of the routing path selection algorithm through two examples. Target and cluster member RFID tags are neglected since they are not involved in the information routing process based on the design. The same simulation parameters are assumed as before.

**Figure 15.**
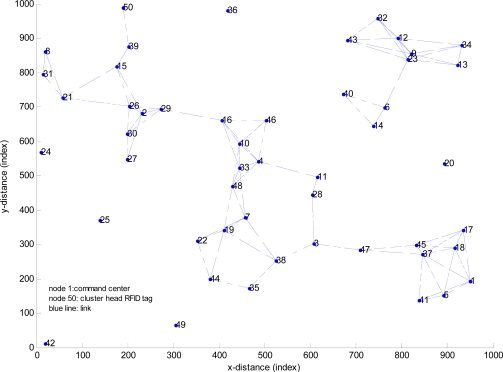
Topology of example 1.

In Example 1, 50 nodes are deployed. Each node represents an RFID tag. Node 1 represents the command center, Node 50 represents the cluster head RFID tag, and the blue line represents the link between two nodes. Weight of the link is quantized to 1 or ∞. The topology of example 1 is shown in Figure 15.

### 3.3. Simulation Results

**Figure 16.**
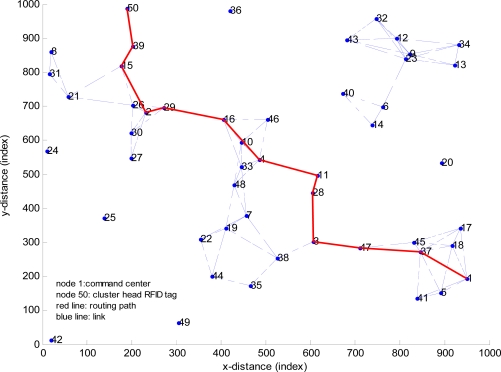
Routing path in example 1.

The target’s information routing path from node 50 to node 1 is depicted by the red line in Figure 16, which is the shortest path between node 50 and node 1.

Nodes on the routing path are:

**Table:** 

path	=	50	39	15	2	29	16	4	11	28	3	47	37	1

and the cost of the path, which is the sum of weights of links on the path is:

**Table:** 

cost	=	12

Example 2 is a small network with ten nodes. Weights of the link are quantized to 5, 10, 20, and ∞. The weights of links are described by the matrix *W*.

(8) $$W=\left[\begin{array}{lllllllllll} \mathrm{index} & 1 & 2 & 3 & 4 & 5 & 6 & 7 & 8 & 9 & 10\\ 1 & 0 & 5 & \infty & \infty & \infty & \infty & \infty & 10 & 20 & \infty \\ 2 & 5 & 0 & 5 & \infty & \infty & \infty & \infty & \infty & \infty & \infty \\ 3 & \infty & 5 & 0 & 5 & \infty & \infty & 10 & \infty & \infty & \infty \\ 4 & \infty & \infty & 5 & 0 & 10 & \infty & \infty & \infty & \infty & \infty \\ 5 & \infty & \infty & \infty & 10 & 0 & \infty & \infty & \infty & \infty & 10\\ 6 & \infty & \infty & \infty & \infty & \infty & 0 & \infty & \infty & \infty & \infty \\ 7 & \infty & \infty & 10 & \infty & \infty & \infty & 0 & \infty & 10 & 20\\ 8 & 10 & \infty & \infty & \infty & \infty & \infty & \infty & 0 & \infty & \infty \\ 9 & 20 & \infty & \infty & \infty & \infty & \infty & 10 & \infty & 0 & \infty \\ 10 & \infty & \infty & \infty & \infty & 10 & \infty & 20 & \infty & \infty & 0 \end{array}\right]$$

Node 1 represents the cluster head RFID tag, and Node 10 represents the command center. Simulation Results:

The routing path from node 1 to node 10 is:

**Table:** 

path =	1	2	3	4	5	10

and the cost of the path is:

**Table:** 

cost =	35.

## 4. Hardware Implementation Considerations

Three important considerations for hardware implementation of the proposed RFID tag structure are: (1) battery life; (2) antennas; and (3) information storage. These are discussed below.

The proposed RFID tags are active RF tags, meaning that they are designed to both receive commands and transmit coded information to other tags in the vicinity. Because an active RFID tag is powered by the internal energy, the lifetime of the tag is mainly dependent on the lifetime of the battery. However, they have built-in circuitry to turn on the transmitter only when they receive “wake up” commands. This extends battery life since higher current is drawn only when powering the transmit chain components. In the passive “sleep” mode, these tags act as simple RF receivers which do not require much battery power for functioning. The sleep mode enables the application to shut down the processor of unused modules, thereby saving power [17]. The receiver, which is kept active to react to an inquiry from the interrogator, therefore determines the shelf life of an active RFID tag. To further reduce the power of the RFID tag receiver two main technologies have been proposed: (1) a passive transceiver or burst switch allowing the tag to remain in a sleep mode until activated with RF energy; and (2) a smart buffer, which allows the controller to remain asleep while an incoming packet is buffered [18]. Use of RFID as the wake-up radio channel has been shown to provide a viable solution due to the low cost and ready off-the-shelf availability of RFID [19]. Furthermore, these circuits are able to wake up an entire neighborhood of nodes if a packet at a particular frequency is received.

These RFID tags must necessarily transmit their information in all directions to ensure that the information is assuredly picked up by other randomly distributed tags in the vicinity. This calls for a low-gain omnidirectional antenna, which is quite advantageous since such an antenna comes in smaller packages, and is therefore consistent with the small size of the tag. The antenna must be small enough to be attached to the tag, have omnidirectional or hemispherical coverage must provide maximum possible signal to the receiver, have a polarization matched to the enquiry signal regardless of the physical orientation of the tag, be robust and cheap [20]. Major considerations in antenna selection are antenna type, its impedance, RF performance when applied to the tag, and RF performance when the tag has other structures around it. Candidate omnidirectional antennas include the dipole and the folded dipole, with bandwidths of 10–15% and 15–20%, respectively [20]. Planar antennas are low cost, simple to manufacture, and have low profile suitable for RFID systems. The most common types of planar antennas for tags are folded dipoles, meander line antennas (MLAs) and spirals [21]. Planar elliptical patch antennas have been shown to be adequate for ultrawideband (UWB) applications in several bands. Such UWB antennas have been used in mobile handset devices with FR4 substrate using standard printed circuit board processes. The availability of high-contrast, low-loss ceramic materials permits significant antenna miniaturization, although they have higher loss characteristics [22]. However, their low profile and UWB operation make them quite attractive for use in RFID tags.

Other considerations include storage requirements. The storage size of the RFID tag depends on the number of possible targets to be monitored, size of the target’s signal, size of the target ID, size of the tag ID, size of the keys, size of the counter, *etc*. Since these are application- and scenario-specific, it is difficult to assess the storage requirements in a general sense. It has been proposed that utilizing local storage of writeable RFID tags for inference and query processing makes the distributed approach a better solution with significantly reduced communication cost [23]. The query state primarily dominates the storage cost, and a larger numbers of queries may challenge the scalability of this approach. An approach to exploit the unique property of prime numbers to encode nodes in the path, and simultaneous congruence values to encode ordering between nodes in the path has been implemented and tested in [24]. The encoding scheme is based on the Fundamental Theorem of Arithmetic and the Chinese Remainder Theorem. Using the proposed path encoding scheme, it was shown possible to efficiently retrieve paths which satisfy the path condition in a query [24]. It is assumed in this paper that adequate storage size is available for proper functioning of the tag.

## 5. Conclusions and Future Work

The design of a covert RFID tag network for target discovery and target’s information routing is presented in this paper. The design and operations of the proposed algorithms are illustrated through examples. Simulation results clearly demonstrate the effectiveness of the design. In the design, we also considered the possible physical layer implementations, and considering that RFID tag’s structure cannot be made very complex, we make the tradeoffs and do not incorporate too many advanced and accurate functionalities for the RFID tags. Although the initial RFID tag network design goals of the research have been achieved in this paper, further theoretical and experimental extensions are possible. For our future work, we plan to investigate more issues to make the design faultless and more practical, such as addressing the holes problem in the RFID tag network during information routing, data traffic and congestion. We will also consider the implementation of a small RFID tag network based on the design.
